# Preparedness of Hospitals in North of Iran to Deal With Disasters

**DOI:** 10.5812/ircmj.4279

**Published:** 2013-06-05

**Authors:** Mohammad Amiri, Reza Chaman, Mehdi Raei, Seiyed Davoud Nasrollahpour Shirvani, Abolhasan Afkar

**Affiliations:** 1 Department of Health Services Management, School of Public Health, Shahroud University of Medical Sciences, Shahroud, IR Iran; 2 Department of Community Medicine, School of Medicine, Shahroud University of Medical Sciences, Shahroud, IR Iran; 3 Department of Biostatistics, School of Medicine, Qom University of Medical Sciences, Qom, IR Iran; 4 Department of General Education, Babol University of Medical Sciences, Babol, IR Iran; 5 Department of Health Services Management, Guilan University of Medical Sciences, Guilan, IR Iran

**Keywords:** Iran, Disasters, Hospital Preparedness

## Abstract

**Introduction:**

Preparedness of hospital has a major impact on their optimal and satisfactory performance. This study aimed to investigate the preparedness of the hospitals to deal with disasters.

**Case Presentation:**

This cross-sectional study was carried in 2011 and all of the hospitals which were located in the northern areas of Iran were investigated through the census method. The data collection instruments were self-administered Managers’ Awareness Questionnaire (40 items) and a 141-item checklist. The mean percentage score of hospitals in management of the unanticipated disasters program in the hospital was good. The mean score of managers’ awareness of the hospital status was moderate. With the increase in managers’ awareness, the preparedness of the hospitals significantly increased (r = 0.73, P < 0.001).

**Conclusions:**

The findings showed the moderate preparedness of the hospitals in the Northern provinces to deal with disasters.

## 1. Introduction

Throughout its ancient history, Iran has witnessed a great many natural and non-natural disasters and in this regard, it is ranked fourth in Asia and sixth worldwide (1). Although only 1% of the world’s population live in Iran, more than 6% of world’s natural disasters occur there (2) and because of being located in Alps–Himalayas Seismic zone, Iran is one of the top 10 countries vulnerable to earthquakes and in fact it is one of the regular victims of quakes (3). Though rare, disasters have destructive effects and they expose the health care system to multitude of patients and casualties. These effects even last long after the crisis (4). Moreover, efficient management of hospitals and healthcare centers can have a constructive role in their optimal and satisfactory performance in crises (5).

Results of a study in hospitals in Tehran indicate the average preparedness of hospitals to deal with disasters (3, 6). In another study carried out in Kermanshah, the preparedness of teaching hospitals of this province to deal with crises have been reported poor (7). With regard to these issues, the present study intended to determine the level of preparedness of some Iranian hospitals in dealing with disasters.

## 2. Case Presentation

In this cross-sectional study, all hospitals (n = 53) of five provinces of Semnan, Golestan, Mazandaran and Gilan were investigated through census method. The data collection instruments included a self-administered Managers’ Awareness Questionnaire (40 items), and a 141 item checklist which includes eight domains of Assessment of Environmental Health Measures (16 items), Managing Unanticipated Events in Hospital (12 items), Planning and Support for Vital Services (17 items), Hospital Educational Program for Dealing with Disasters (18 items), Planning Safety of Equipment and Hazardous Materials in Disasters (26 items), Reducing Construction Dangers (8 items), Planning for Evacuation and Field Treatment (20 items), Planning for Necessary Medical and Nonmedical Equipment and Consumables (16 items) and 8 general items which were completed by the managers of the hospitals(self –assessment) based on the documents. The reliability of the questionnaire was examined through test-retest method and Pearson correlation was found to be 0.91. SPSS 13 was used to analyze the data through Man-Whitney and Kruskal Wallis tests. To investigate the correlation, the Pearson correlation was used. In this study, the confidence level was 95% and the significance level was 0.05.

41.5% of the respondents had bachelor’s degrees, 17% had master’s degrees and 39.7% had PhD or were general practitioners. 45.3% of respondents majored in nursing, 37.7% majored in medicine, and 17% majored in other fields such as management of healthcare services, environmental health or laboratory sciences. 64.2% of respondents had work experience less than 10 years, 32.1% had 10-20 years of experience, and 3.8% had over 20 years of experience. 58.5% of the managers stated that they had been trained on disaster management and 96.2% of managers stated their need for educational and refreshment courses in this regard. In general, 35.8% (n = 19) of hospitals had weak preparedness, 54.4% (n = 25) had moderate preparedness, and 9.4% ( 5 ) had good preparedness. Mean percentage score of hospitals in the domain of managing unanticipated events in hospitals was good, and in scopes including planning support for vital services, safety of equipment and hazardous materials in disasters, necessary medical and nonmedical equipment and consumables was moderate and in other scopes including hospital educational program, environmental health measures, reducing construction dangers, and evacuation and field treatment was weak (Table 1).

**Table 1.** Preparedness of Hospitals to Deal With Disasters

Scopes of Preparedness	Mean ± SD
**Managing Unanticipated Events in Hospital**	77.36 ± 22.25
**Hospital Educational Program for Dealing with Disasters**	44.76 ± 26.20
**Support of Vital Services**	72.91 ± 19.65
**Environmental Health Measures**	47.46 ± 26.77
**Safety of Equipment and hazardous Materials in Disasters**	61.75 ± 15.55
**Reducing Construction Dangers**	40.56 ± 29.4
**Evacuation and Field Treatment**	42.92 ± 21.43
**Necessary Medical and Nonmedical Equipment and Consumables**	65.09 ± 28.26
**General Preparedness**	56.88 ± 15.12

The mean score of managers’ awareness of hospital conditions was 41.89 ± 9.12 which shows the moderate awareness of the managers. With the increase in managers’ awareness scores, the preparedness of hospitals significantly increased (P < 0.001, r = 0.73). In general, 35.8% of hospitals had poor preparedness and more than half of the managers (85.7%) were not adequately trained in disaster management. No significant difference was observed between the average preparedness score and work experience of the manager (P = 0.21), gender (P = 0.63), manager’s education (P = 0.17) and manager’s field of study (P = 0.06) (Table 2).

**Table 2.** Relationship Between Hospital Preparedness and Some Variables

	No.	Mean ± SD	P value
**Manager’s Experience**			
< 10 years	34	73.03 ± 18.15	0.27
10-20 Years	17	82 ± 23.01	
> 20 years	2	66.5 ± 24.74	
**Manager’s gender**			
Male	44	76.09 ± 19.41	0.63
Female	9	73.56 ± 24.45	
**Manager’s education**			
Bachelor’s and less	23	76.61 ± 18.1	0.17
Master’s	9	86 ± 25.25	
PhD or GP	21	70.19 ± 18.87	
**Manager’s Field of Study**			
Nursing	24	76.71 ± 22.27	0.06
Medicine	20	68.2 ± 16.94	
Management	2	87 ± 4.24	
**Others**	7	90.14 ± 14.66	

There was a significant relationship between training on disasters and manager’s awareness (P = 0.007), but no significant relationship was observed between training and general preparedness of the hospital (P = 0.8).

## 3. Discussion

According to findings of this study; 15.1% of managers had no training on dealing with disasters. Other studies reported the number of untrained managers to be 6.7% and 13% and in some areas even 47.6% and 73% (3, 6, 8, 9). Neither were there significant relationships between the preparedness of the hospital and the manger’s age, experience in management, education, field of study and training to deal with disasters. Hosseini also reported similar results (7). Preparedness of hospitals in managing unanticipated events in hospital and supporting vital services was assessed to be rather good which is close to the findings of other studies (6, 10). In this study, the mean percentage of hospital preparedness in reducing the construction dangers was less than that of other domains. The poor building status of hospital in this domain in all other studies carried out in Iran is consistent with this finding (6, 8, 10). Hospital preparedness in the domain of educational planning on dealing with disasters was weak. This is consistent with the findings of other studies (7, 8, 11). However, this finding is not consistent with the findings of the study by Top and colleagues in Turkey and Cliff in the United States, which showed the good preparedness of hospitals in this domain (11, 12).

## 4. Conclusions

The findings indicate that the hospitals in the northern provinces of the country, have moderate preparedness to deal with disasters. Organizing educational programs on dealing with disasters, developing educational programs for hospital personnel and strengthening hospital buildings and organizing practice maneuvers can play an effective role in increasing hospital preparedness to deal with disasters.
